# Longitudinal effects of affective distress on disease outcomes in rheumatoid arthritis: a meta-analysis and systematic review

**DOI:** 10.1007/s00296-024-05574-9

**Published:** 2024-05-22

**Authors:** Melissa Sweeney, Maryam A. Adas, Andrew Cope, Sam Norton

**Affiliations:** 1 Health Psychology Section, Institute of Psychiatry, Psychology and Neuroscience, King’s College London, 5th Floor, Bermondsey Wing, Guy’s Hospital, Great Maze Pond, London, SE19RT UK; 2 Centre for Rheumatic Diseases, King’s College London, London, UK

**Keywords:** Psychological stress, Rheumatoid arthritis, Prospective studies

## Abstract

Patients with rheumatoid arthritis have higher rates of mental health conditions compared to the general population. It is believed that affective distress and rheumatoid arthritis have a bi-directional relationship. This review will examine the associations between affective distress and rheumatoid arthritis outcomes over time. Several disease outcomes are included covering disease activity, function, and disability to provide a broad picture of the various ways patients are impacted. A quality assessment was also conducted. There were 71 studies included in the review. Three measures (disease activity, disability, and mortality) had enough data to complete meta-analyses of odds ratios or hazard ratios. The outcomes included were disease activity, tender joint count, swollen joints, pain, physician global assessment, patient global assessment, physical disability, acute phase reactants, stiffness, fatigue, work disability, and mortality. Numerous measures were included for most of the outcomes due to the variability across studies of measures used. Patients with affective distress had lower rates of remission according to the DAS-28, greater disability, and higher mortality. All of the outcomes covered had studies with mixed results, but swollen joint count, tender joint count, patient global assessment, and physician global assessment had the strongest evidence that they were associated with mental health longitudinally. The relationships between affective distress and disease outcomes are complex and vary depending on the measures. Overall, the effects fade over time. It is important for clinicians to be aware of the differing manifestations of the relationship between affective distress and rheumatoid arthritis outcomes.

**Supplementary Information:**

The online version contains supplementary material available at 10.1007/s00296-024-05574-9.

## Introduction

Rheumatoid arthritis (RA) is a chronic progressive autoimmune inflammatory disease that primarily affects the joints. It is approximately twice as common in females compared to males [1], with a combined prevalence rate of ~ 1% in the adult population [2]. The primary feature of RA is joint inflammation and swelling (synovitis), which typically causes pain and reduced function, which can greatly impact on the patient’s quality of life. Where inflammation is not adequately controlled, persistent inflammation can lead to irreversible joint damage, which can further impact on function and quality of life [3, 4].

Patients with RA have higher rates of affective distress, namely depression and anxiety, than the normal population [5, 6]. Estimates indicate that between one-fifth to one-third of RA patients have comorbid depressive symptoms, indicative of depressive disorder [7–9]. Furthermore, patients with affective distress and RA have worse disease outcomes in both the short-term and long-term, even after accounting for disease severity [10, 11].

Affective distress and RA may have a bi-directional relationship [9, 12]. RA is believed to impact depression symptoms through various mechanisms such as arthralgia and disability associated with RA resulting in depression [13] while depression may affect RA symptoms through illness-related cognitions [14, 15]. Recent findings also suggest that depression may at least in part be an extra-articular manifestation of RA-related inflammation [9, 16–18]. Mechanisms for affective distress affecting RA outcomes include altered pain perception [19] and shared inflammatory processes [20]. While there has been less research on the biological mechanisms linking anxiety and rheumatoid arthritis, anxiety has also been found to be higher in RA patients [21]. Given the overlap between RA and affective distress causes and processes, they may also negatively affect one another in a downward spiral, leading to both worse affective distress and RA outcomes.

Since the relationship between affective distress and RA seems to be complex and inter-dependent, longitudinal studies are needed to disentangle the temporality and causality of the effects of affective distress on disease outcomes. However, most of the existing literature has been cross-sectional, focusing on associations between the two factors [22, 23]. Furthermore, drawing conclusions across studies has been difficult because numerous different outcome measures of RA have been used, ranging from biological markers to subjective ratings. Similarly, differing measures of depression or anxiety have been used, focusing on different aspects or symptoms.

The aim of this review is to synthesize the extant literature on longitudinal studies of affective distress and clinical outcomes in adults with rheumatoid arthritis, in order to determine if affective distress is associated with worse clinical outcomes over time.

## Methods

### Identifying publications

The search strategy followed the PICO framework using key terms to conduct searches of the following computerized databases: MEDLINE, PSYCinfo, PSYCarticles, Embase, Cochrane Library, Web of Science, and SCOPUS. Grey literature was identified by searching System for Information on Grey Literature, OpenGrey, and EthOS. Finally, a manual search of reference lists from included studies was conducted. The search strategy included unpublished and published articles or conference abstracts. The timeline included articles from 1998 to 2023.

Keywords were used according to each database’s formats and included the following words or phrases: “depression/depressive symptoms,” “anxiety,” “mood,” “psychological/emotional distress,” “affect/affective disturbance, “rheumatoid arthritis,” “longitudinal,” “prospective,” “cohort,” “retrospective”. An example of the combination of a keywords combination used in the search strategy for outcomes is: “disease activity” OR “clinical outcomes” OR DAS28 OR “DAS 28” OR DAS-28 OR “disease activity score” OR HAQ OR “health assessment questionnaire” OR VAS OR “visual analog scale” OR “pain” OR SJC OR “swollen joint count” OR TJC OR “tender joint count” OR CRP OR “C-reactive protein” OR stiffness OR CDAI OR “clinical disease activity index” OR SDAI OR “simple disease activity index” OR “american college of rheumatology” OR ACR. With Wildcards, the search example becomes: “disease activity” OR “clinical outcomes” OR DAS28 OR “DAS?28” OR “disease activity score” OR HAQ OR “health assessment questionnaire” OR VAS OR “visual analog scale” OR “pain” OR SJC OR “swollen joint count” OR TJC OR “tender joint count” OR CRP OR C?-RP OR “C-reactive protein” OR stiffness OR CDAI OR “clinical disease activity index” OR SDAI OR “simple disease activity index” OR “american college of rheumatology” OR ACR.

### Inclusion/exclusion criteria

Studies which met the following criteria were included: (1) Participants were adults 18 years old and over with rheumatoid arthritis and (2) the studies were longitudinal observational studies, including prospective, cohort, and retrospective studies or randomized controlled trials treated as observational studies where depression, depressive symptoms, anxiety, or psychological or affective distress is considered as a prognostic marker, or predictive marker of treatment efficacy. Studies in any language were included where it was possible to translate the paper. There were no date restrictions. Exclusion criteria were: (1) studies with participants under age 18 (2) studies that were not longitudinal or not including the mental health conditions of the inclusion criteria.

### Outcomes

The primary outcome of interest was the DAS 28; however, secondary outcomes will include the core criteria outlined by OMERACT (Outcome Measure in Rheumatology): Tender Joint Count (28 count TJC), swollen joints (28 count SJC), pain [Visual Analog Scale (VAS), Arthritis Impact Measurement Scales (AIMS)], physician global assessment [Evaluator Global Assessment (EGA)], patient global assessment [Patient Global Assessment (PGA), VAS], physical disability [Health Assessment Questionnaire (HAQ)], acute phase reactants [C-reactive protein (CRP), Erythrocyte Sedimentation Rate (ESR)] [24]. Radiographs were not included since that measurement is used only for studies of at least one year. OMERACT outcomes that were not part of the core measures, but were included were: Stiffness (Duration prioritized) and fatigue (VAS).

Additional outcomes outside of OMERACT that included were: Disease Activity (Simple Disease Activity Index (SDAI), Clinical Disease Activity Index (CDAI), Work disability/impairment, and Mortality.

### Publication screening

One reviewer (MS) screened the titles/abstracts of all of the studies identified in the search according to the inclusion/exclusion criteria. A second reviewer (MA) independently screened 10% of the full texts that were included. Agreement at the full text stage was achieved in 100% of the papers. A flow chart of the screening process is found in Supplementary Material Fig. S1.

### Data extraction

One reviewer (MS) extracted data from the included studies using a pre-designed form for data extraction. Information was extracted about (1) Sample characteristics (e.g. mean age, proportion female) (2) Sample size at each time point, and flowchart of participation (i.e. attrition) (3) Eligibility (4) Study rationale and aims (5) Depression/mental health measures (6) Study design (7) Outcome measures categorised by type (e.g. disease activity, symptom) (8) Types of analysis, including covariates adjusted for (9) Dates of follow up 10) Effect estimate and its precision (i.e. standard error), or sufficient data to calculate precision (e.g. standard deviation) (11) Publication date (12) country of publication and (13) cohort. In cases where the necessary data was not given in a publication, the authors were contacted.

### Quality assessment

Risk of bias was assessed using the Quality In Prognosis Studies tool. Grading of Recommendations, Assessment, Development and Evaluation (GRADE) will be used to evaluate the strength of the body of evidence.

### Statistical analysis

Meta-analysis of each outcome measure with sufficient data and graphs was conducted in STATA 16.0. Due to there being many different outcomes and methodological heterogeneity, meta-analysis was only done for the outcomes measured dichotomously where possible, then a narrative synthesis was done using an effect size based vote counting approach across all studies. Random-effects models were used to estimate the weighted effect size for each outcome measure. For mortality, the weighted hazard ratio was also calculated. The I^2^ statistic was also calculated to determine the level of heterogeneity among the effect sizes. Lastly, forest plots were generated to display the weighted effect size.

## Results

In total, there were 71 studies included in this review (Supplementary Table 1). The most common outcome measure was the DAS28, with 31 studies reporting results for it (Supplementary Table 2). Disability measures, primarily the HAQ, were also frequently used. The remaining outcomes had fewer studies, but many were still prevalent in the literature. The studies also represented a wide number of countries, though studies from the UK and USA were most common in the sample. A majority of the studies also had large sample sizes, which would have made them better able to detect effects. A quality assessment using the Newcastle-Ottowa Scale is presented in Supplementary Table 3.

### Mortality

**Fig. 1:**
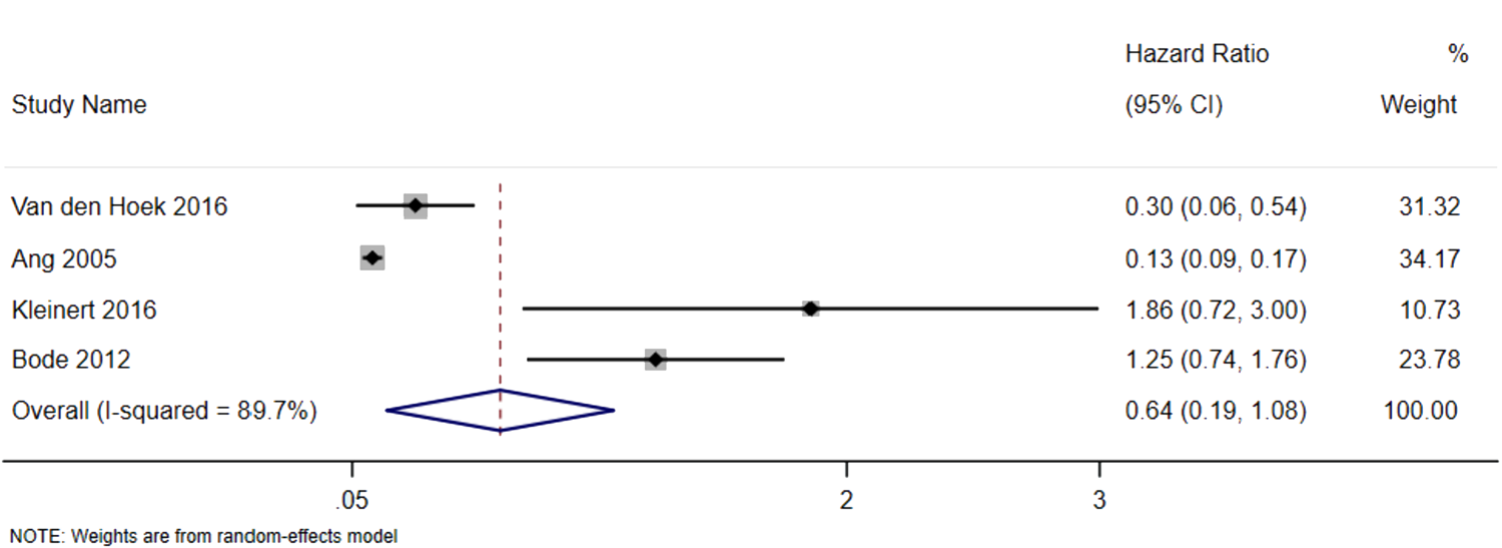
Random-effects hazard ratio forest plot for mortality

Meta-analysis of four studies on mortality showed a pooled effect estimate of 0.64 (95% CI: 0.19, 10.8). The pooled hazard ratio estimate was 2.98 (95% CI 1.49–5.97) (see Fig. 1). These indicate that patients with RA who have comorbid depression have higher risk of mortality than non-depressed patients. However, since the I^2^ statistic indicates high heterogeneity (I^2^ = 90.3%), these results should be interpreted with caution.

### Disease activity

There were 34 studies investigating the relationship between affective distress and disease outcomes over time, including the DAS, CDAI, and SDAI. Due to heterogeneity in the methods used and range of outcomes it was only possible to undertake meta-analysis for DAS remission.

**Fig. 2:**
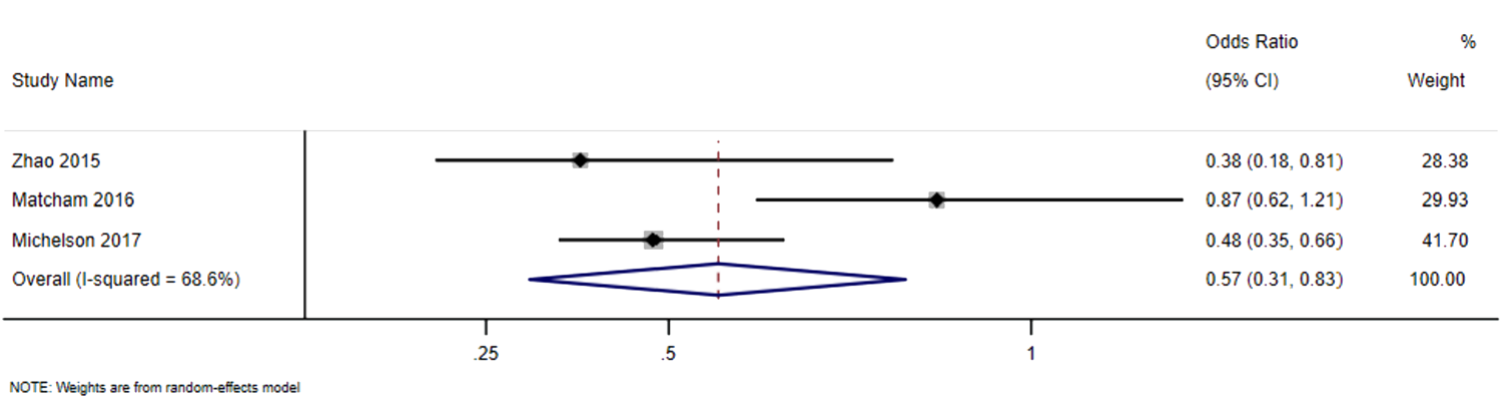
Random effects odds ratio forest plot for DAS remission

Meta-analysis on the odds ratio for remission using the DAS showed a pooled effect estimate of 0.57 (95% CI 0.51–0.83). The meta-analysis generated a pooled odds ratio of 1.77 (95% CI 1.36–2.29) (Fig. 2). These results indicate that patients with affective distress have a lower chance of reaching remission compared with those without affective distress. These results should be interpreted with caution as the predictors of the odds ratio were mixed between depression and anxiety and the heterogeneity for the analysis is high (I^2^ = 68.6%).

Across all 34 studies, just over half found a positive effect where affective distress was associated with disease activity, though there were several nuances and nearly half of the studies had an unclear effect. The amount of time for the follow-up ranged from 13 to 104 weeks so it is perhaps not surprising that the findings would be conflicting over such a large range. However, the general trend indicates that the effect fades over time. One study with more frequent follow ups detailed this trend, with the effect only being significant at 13 weeks while the remaining follow ups for just over a year no longer showed an effect [25]. Another factor which influenced the results was the type of affective distress. Distinguishing between depression and anxiety brought mixed results for some studies, though no definitive overall trend was discernable. Similarly, the scales used to measure affective distress seemed to affect the results as well, with some studies using scales such as the SF-36 or EQ5D, which were not originally designed specifically for affective distress. The results of studies with these scales were mixed but mostly indicated that scales which are more specific to mental health were linked with later effects on disease outcomes whereas more general scales were not [11, 26, 27]. Overall, the findings indicate that affective distress indeed affects disease outcomes over time, though the amount of time this effect lasts is undetermined but likely fades over time.

### Somatic symptoms

#### CRP

Three studies examined the effects of affective distress on CRP longitudinally. None of the studies found clear significant associations, even though one of them followed participants for 1 year. The inconclusive results could be due to smaller sample sizes in the studies that did not find significant results, whereas the CORRONA study had 12,445 participants so it had more power to detect differences between groups. Furthermore, the differences in log CRP were small so it is likely that a study would need high power in order to detect significant differences. Distinguishing between depression versus anxiety did not change these inconclusive results since the study which distinguished between them had insignificant findings for both conditions.Thus, while affective distress did not appear to be clearly associated with elevated CRP, it's possible the differences were too small to be detected.

#### ESR

Of the 8 studies which measured ESR, the relationship with affective distress was unclear for all of them. However, one study which used various measures of affective distress included history of depression rather than only current psychological distress and found that significantly predicted ESR [26]. Another study found an association between psychological distress and ESR at 6 months [28]. These were both the shortest-term studies included. This points towards the possibility that the effects of psychological distress on ESR exist but wane over time. However, the number of studies with similar timepoints for the short vs long-term is small so it is difficult to conclude with much certainty that the effect on ESR diminishes with time. When depression and anxiety were considered separately, the results remained unclear since the findings were insignificant for both conditions. Greater consistency in the measurement of affective distress, its subcomponents, and the time periods measured in future studies could clarify the relationship.

#### TJC

There were 7 studies found that investigated affective distress and TJC. The scales used and the results were mixed, but 5 of the 7 studies found a significant relationship between affective distress and TJC. The type and severity of affective distress of the studies seemed to define the findings. For example, one study separated affective distress by severity and found those with more severe depression had worse TJC scores [29]. For those studies which measured depression versus anxiety, rather than affective distress more broadly, many more focused on depression. There was only one study which examined anxiety alone and its results were consistent with the depression results [29]. While there is nuance in the severity and type of affective distress in its effect on TJC, the studies overall indicate that affective distress indeed worsens TJC.

#### SJC

There were 7 studies which investigated the relationship of affective distress on SJC. Similar to TJC, 5 of the 7 studies indicated an effect of affective distress on SJC. This included studies which reported mean scores for depressed vs not depressed patients, but did not report their statistical significance. However, the differences were small (mostly 0.1 or fewer points difference) between the groups so the results also seem to point towards affective distress having less of an effect on SJC compared with other outcomes included in this review. In the three studies that distinguished between anxiety and depression, the direction of the effect was different in one, but it was very small and insignificant [25]. The remainder of studies that distinguished between anxiety and depression did not have any significant differences [30, 31]. The other studies either used only depression or scales such as the SF-36, which measures affective distress more broadly. In terms of timelines, studies lasted a year or more so it is also possible that the effect is short and fades by 1 year so it was not detected by these studies.

#### EGA

Four studies examined the effect of affective distress on EGA. Most, 3 out of 4, found a significant association between poor mental health and higher EGA scores. Two studies distinguished between severity levels of depression and found greater depression was associated with worse EGA scores [26, 32]. Many of the studies reported only the means without testing for statistical significance so conclusions based on these are limited. However, overall the studies seem to indicate that affective distress is associated with worse EGA scores, with increasing affective distress associated with increasing EGA. The studies either focused on depression alone or affective distress rather than distinguishing between anxiety versus depression, but there was no discernable difference in the results based on these measures. The studies lasted 1–2 years so it appears that the effects of affective distress on EGA may also be longer lasting, compared with other outcomes included in this review.

#### PGA

There were 2 out of 3 studies which indicated a link between affective distress and PGA. The remaining study reported means over time so the direction of effect was unclear. However, the results across studies showed that the greater the depression scores, the greater the PGA scores. While the variation in statistical methods used between studies makes it difficult to draw any conclusions beyond the single study that tested for an association, they did show increased PGA when affective distress was increased. There was a mix of studies that distinguished between anxiety or depression and those that combined them into affective distress, but the results were not clarified along these lines. Similar to EGA, the studies also lasted 1–2 years, during which time any associations could fade anyway.

### Pain

There were 12 studies investigating pain and affective distress. Just under half of the studies, or 5 of the 12, found a relationship between these two factors found a significant relationship. This may be partly due to the outcomes measures being mixed between the VAS, AIMS, and MPQ, though most reported the VAS. Overall, while pain and affective distress were found to be significantly related in nearly half the studies, the results may be mixed due to the relationship being more complex than could be detected in the existing studies, given that pain is one of the most subjective measures included in this systematic review. For example, previous studies have shown the threshold of pain to be lower in patients with depression so the relationship between pain and affective distress may be interdependent so studies aimed at teasing apart these concepts could better explain the mixed findings [33]. Most studies focused on affective distress, but some studied depression alone, while only one studied anxiety alone. There was a significant relationship between anxiety and both pain outcome measures, which aligns overall with the studies that used affective distress or depression [34].

### Stiffness

There was only one study that examined psychological distress and stiffness and revealed a complex relationship [35]. Patients were separated into trajectories of affective distress, but all of them showed decreases in early morning stiffness by the end of 3 years. However, the amount of decrease varied based on the level of distress. Those who ended with higher distress had similar stiffness scores by the end of 3 years, regardless of their starting point. Although this does not support the conclusion that earlier psychological distress has long-lasting effects on stiffness, it does strengthen the conclusion that in the immediate or short-term, affective distress is closely related with stiffness.

### Fatigue

The results for the effect of affective distress on fatigue were mixed in that half of the studies indicate that depression is significantly associated with fatigue while the remaining studies did not find any significant relationship. This was not entirely clarified by distinguishing between depression, anxiety, or affective distress since there was only one study focused on affective distress while the rest studied depression, but the results of the study for affective distress were insignificant. However, it is possible this is an important distinction for fatigue since it could be more linked with depression than anxiety or affective distress more generally. The studies which were significant looked at fatigue scores within a few months of the depression scores whereas those that were not significant were examining scores a year or more later. These results could indicate that the effect of affective distress on fatigue is significant, but only in the short-term.

### Disability

There were 23 studies which examined affective distress and its association with disability long-term. While the majority (13) of the studies did not have conclusive results, several studies did find a significant association between affective distress and later disability, with only two studies finding no relationship. The outcome measures used for disability were the most diverse of all the outcomes included in this review, covering HAQ, AIMS, ADL, GARS, MHAQ, SF-36, and Short-term disability days, so the variety may explain some of the mixed results. The affective distress measures were also diverse but did not yield different results for those which focused on anxiety versus depression versus affective distress. Furthermore, the follow-ups were over the longest time periods, compared with other outcomes, with only two studies including follow-ups at less than a year. Thus, the effects could have been missed or less pronounced due to the longer time between baseline and the follow-ups. While it seems likely that affective distress is related to disability over time, the conclusion is tentative due to the inconsistency of the measures and long time periods.

**Fig. 3:**
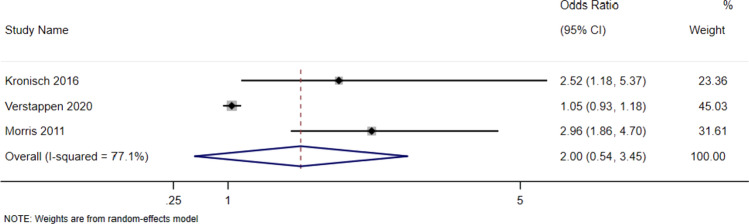
Random effects odds ratio forest plot for HAQ

Meta-analysis of depression on the odds ratio of disability, using the HAQ showed a pooled effect estimate of 1.99 (95% CI 0.54–3.45). The pooled odds ratio was 7.37 (95% CI 1.72–31.64), indicating that patients with depression have a greater risk of disability. The heterogeneity was high (I^2^ = 77.1%) so these results should also be interpreted with caution (see Fig. 3).

### Work disability

There were only 2 studies which examined the effect of emotional distress or depression on presenteeism with conflicting results with one finding a negative effect and the other inconclusive. The study with an effect in the negative direction used anxiety, which may indicate patients try harder to keep up their work to prevent further anxiety so the type of distress could determine the direction. A third study also examined the effect of affective distress on productivity, which could be considered closely related and similarly found an effect in the negative direction, but the measure was psychological distress more generally. There were also a couple additional studies that examined work absence, including absenteeism, sick leave, and leaving the job. One study showed slightly elevated chance of sick leave for those who were depressed while the other study found a higher 3.2 risk ratio for sick leave for anxious patients [36], indicating that the type of emotional distress may also affect outcomes for work disability, similar to other outcomes in this review. The mixed results could also be due to differing scales, different severities of affective distress, or the small number of studies. Distinguishing between anxiety, depression, or affective distress did not change these results since the findings were mixed among these aspects of mental health. Lastly some aspects of work disability, such as presenteeism, could be subjective so patients who are depressed or anxious may have less accuracy in reporting their productivity, which could influence the results.

## Discussion

The meta-analyses and narrative review present an overall picture that affective distress impacts RA patients not just in their day-to-day symptoms, but over their lives through work disability and earlier mortality. While the studies were quite mixed in the reported outcomes and measures of affective distress, they seem to indicate that affective distress has an impact, but the specific outcomes which are impacted vary such that not all outcomes are equally affected. These varied results may be helpful for clinicians to understand the different presentations that they may see in clinics.

The differences between the results of the studies also highlighted the subjectivity of many of the measures. For example, TJC was significantly related with affective distress in most studies, whereas SJC, which could be considered more objective, was only found to be linked in two studies. In contrast, the most objective measures, such as CRP and ESR for inflammation, only showed weak evidence for a relationship with affective distress. This pattern is evident overall as well, with more subjective measures showing more associations while more objective measures showing fewer associations. The implications of this highlight the inconsistencies across clinics and measures and potential improvements possible in research by standardization.

However, it may be a more complex situation since the inflammatory markers had opposing results depending on the follow-up timing. It could be that they are affected by affective distress, but only in the short-term. Mood also varies over time, even within the day, so it may be more complex to collect accurate measurements of mood and disease outcomes at the right timepoints [37]. Alternatively, depressive mood may cause patients to report worse symptoms as it could influence their perception of the symptoms. However, it is also possible that the objective ratings are missing something since they may be done by an outside observer or provide only one piece of information.

Additionally, the present study not only used many different outcomes, but also many varied affective distress measures, ranging from history of depression, to anxiety only, to mixed affective distress, to severe depression only. There were multiple scales used. Despite these differing methods, there were sometimes differing results for depression versus anxiety where they could be distinguished, but there was not enough evidence in this review to adequately distinguish between their effects. However, evidence so far shows that they may share some overlapping biological pathways, but not all [38]. Adopting more specific and universal measures could make it easier to draw conclusions due to more consistency or specificity. There are also behavioral and cognitive differences that could distinguish their effects on physical outcomes. Future studies should be designed with awareness of the possible different effects so that they can be accurately captured.

This study had the benefit of a large size of many studies covering many different outcomes. However, there were also limitations, such as the large variance in outcome measures used which prohibited meta-analysis for most measures. The follow-up times also made it difficult to accurately assess the true effects of affective distress since these effects could have been missed due to the follow-ups occurring too soon or too late. Similarly, while the time range of the studies included was large, attitudes towards mental health and treatments for RA could have shifted during that time.

Although there are many differences in the details of the studies, taken as a whole, they appear to confirm that affective distress affects physical symptoms over time. This has the implication of suggesting greater attention is needed to the mental health of RA patients in clinical care. Greater consistency across studies in the future will help further clarify these relationships. These results can be useful for patients and clinicians to better understand the complex and long-lasting relationship between affective distress and physical RA symptoms.

**Table:** 

Author	Year	Cohort	Country	Study Design	Sample Size	Weeks Follow up	Mean age	% Female	Measure
Zhao [39]	2015		UK	Observational	126	52	Not reported	Not reported	DAS28, HAQ,
Matcham [40]	2015		UK	Observational	385	52	59	81	DAS28, HAQ, ESR, TJC, SJC, PGA
van den Hoek [41]	2016		Netherlands	Observational	882	156	59	72	HAQ, EGA, Mortality
Miwa [42]	2015		Japan	Intervention	333	26	Not reported	Not reported	SDAI
Brown [43]	1990		USA	Observational	387	182	53	75	VAS pain
Kronisch [44]	2015	SERA	UK	Observational	578	52	61	65	HAQ
Corominas [45]	2014		Spain	Observational	120	104	52	87	DAS28
Gwinnutt [46]	2019	RAMS	UK	Intervention	463	52	Not reported	68	Work disability (work leave, sick leave, presenteeism)
Rathbun [47]	2016	CORRONA	USA	Intervention	1820	52	58	75	CDAI
Matcham [31]	2016		UK	Observational	56	52	54	79	DAS28, ESR, TJC, SJC, PGA
Kronisch [48]	2016	SERA	UK	Observational	1140	52	61	65	HAQ
Miwa [49]	2017		Japan	Retrospective intervention	232	26	55	71	HAQ
Ang [50]	2005		USA	Observational	1290	939	57	73	Mortality
Parenti [51]	2016		USA	Retrospective intervention	4064	26	Not reported	Not reported	DAS 28, CDAI
Cui [52]	2015	OBRI	Canada	Observational	2305	26	Not reported	Not reported	DAS28, SDAI
Corominas [53]	2019		Spain	Intervention	2305	26	Not reported	Not reported	FACIT-F
Michelsen [54]	2017	NOR-DMARD	Norway	Intervention	1326	26	54	75	DAS28, CDAI, SDAI
Kleinert [55]	2016		Germany	Observational	764	261	54	80	Mortality
Hider [56]	2009		UK	Intervention	160	52	56	72	DAS28
Bode [57]	2012		USA	Observational	530	255	60	84	Mortality
Leblanc-Trudeau [58]	2015	EUPA	Canada	Intervention	275	182	61	63	SDAI
McFarlane [59]	1988		Australia	Observational	30	156	53	66	DAS28
Norton [60]	2011	ERAS	UK	Observational	784	156	57	67	DAS28, HAQ
Michelsen [61]	2017	NOR-DMARD	Norway	Intervention	1326	26	54	75	DAS28, CDAI, SDAI
Kuijper [25]	2018		Netherlands	Intervention	281	65	53	68	DAS28, ESR, SJC
Feldthusen [62]	2016		Sweden	Observational	65	52	54	74	VAS fatigue
Gonzalez-Lopez [36]	2013		Mexico	Observational	123	52	44	73	Sick leave
Tanaka [63]	2019		Japan	Intervention	377	104	Not reported	Not reported	WPAI
Doeglas [64]	2004		Netherlands	Observational	264	156	53	65	GARS
Verstappen [65]	2007		Netherlands	Intervention	112	52	49	68	HAQ
Hommel [66]	1998		USA	Observational	42	52	53	81	MHAQ
Fifield [67]	2001		USA	Observational	415	417	58	83	VAS fatigue
Looper [68]	2011	McEAR	Canada	Retrospective observational with history of depression	104	Medical History	54	61	HAQ
Nugaliyadde [69]	2017		UK	Intervention	13	51	61	73	DAS28
Chung [70]	2013		Australia	Observational	114	261	Not reported	Not reported	DAS28, HAQ
England [71]	2015	BRAGGS	UK	Intervention	1847	26	58	76	DAS28
Sergeant [72]	2015	RAMS	UK	Intervention	460	26	60	73	DAS28
Morris [73]	2011	UCSF RA panel	USA	Observational	1115	939	55	80	HAQ
Van Den Hoek [74]	2013		Netherlands	Observational	882	574	59	72	HAQ, SF-36
Bechman [11]	2018	OPTTIRA	UK	Intervention	97	52	57	74	DAS28
McFarlane [75]	1987		Australia	Observational	40	156	53	80	DAS-28
Odegard [34]	2007	EURIDISS	Norway	Observational	238	521	52	74	VAS pain
Vriezekolk [76]	2010		Netherlands	Intervention	73	6	53	72	AIMS, VAS pain
Sergeant [77]	2016	RAMS	UK	Intervention	1050	26	59	70	DAS28
Sergeant [78]	2018	RAMS	UK	Intervention	1656	26	59	67	DAS28
Casalla [79]	2013	CONAART	Argentina	Observational	237	52	49	84	DAS28
Cook [80]	2016	NOAR	UK	Observational	868	261	56	66	SJC
Treharne [81]	2008		UK	Observational	189	52	56	74	VAS fatigue
Dobkin [82]	2013		Canada	Observational	248	52	59	63	MPQ-SF
Leggett [83]	2017	RAMS	UK	Intervention	308	52	52	66	WPS-RA
Dyball [30]	2018	BRAGGSS	UK	Intervention	2919	26	57	76	DAS28, CRP, TJC, SJC
Schieir [84]	2016	ERA	Canada	Intervention	1595	52	54	72	DAS28
Parker [85]	1992		USA	Observational	80	26	61	0	SJC
Norton [60]	2011	ERAS	UK	Observational	784	261	57	67	HADS, ESR, VAS pain, TJC, SJC, Stiffness
Matcham [40]	2015	CARDERA	UK	Intervention	467	104	54	68	HAQ, ESR, SJC
Iannaccone [86]	2016		USA	Observational	264	104	57	83	DAS28, MHAQ, CRP
Schieir [87]	2009	McEAR	Canada	Observational	320	26	57	69	MPQ, SJC
Rathbun [88]	2013	CORRONA	USA	Observational	4250	104	Not reported	Not reported	CDAI, HAQ, CRP, ESR, VAS pain, TJC, SJC, EGA, PGA
Rathbun [89]	2015	CORRONA	USA	Observational	12,445	104	58	73	DAS28, CDAI, HAQ, CRP, ESR, TJC, SJC, EGA, PGA
El Miedany [90]	2013		Egypt or UK	Observational	264	156		68	DAS28, TJC,
Uhlig [91]	2000	EURIDISS	Norway	Observational	238	261	51	74	AIMS
Li [92]	2019	Truven Health Marketscan databse	USA	Observational	46,700	52	52	78	Short-term disability
Matcham [32]	2014	CARDERA	UK	Intervention	467	104	54	68	DAS28, HAQ, ESR, VAS pain, TJC, SJC, EGA
Van Den Hoek [93]	2013		Netherlands	Observational	882	574	59	Not reported	HAQ, SF-36
Matcham [94]	2014	CARDERA	UK	Intervention	379	104	54	68	SJC
Crotty [95]	1994		Australia	Observational	75	191	42	100	HAQ
Overman [28]	2011		Netherlands	Intervention	545	26	56	69	TJC
Matcham [26]	2018	BSRBR-RA	UK	Intervention	18,421	52	56	76	DAS28, ESR, SJC, EGA
Euesden [27]	2017	CARDERA	UK	Intervention	520	104	55	69	DAS28, HAQ, ESR, VAS pain, TJC, SJC, EGA
Smedstad [96]	1997	EURIDISS	Norway	Observational	238	104	52	73	HAQ, VAS pain
Karpouzas [97]	2017	UCLA RA cohort	USA	Observational	156	52	52	89	HAQ

### Supplementary Information

Below is the link to the electronic supplementary material.

Supplementary file1 (DOCX 220 KB)

## Data Availability

Data is available from authors upon request.
