# Supplementary material for: Longitudinal effects of affective distress on disease outcomes in rheumatoid arthritis: a meta-analysis and systematic review
Source: Rheumatol Int. 2024 May 22;44(8):1421–33. doi: 10.1007/s00296-024-05574-9 (PMC11222178; doi:10.1007/s00296-024-05574-9)
Supplement: Supplementary file 1 — Supplementary file1 (DOCX 220 KB) [file 296_2024_5574_MOESM1_ESM.docx]

**Supplementary Material**

Figure S1. Flowchart of screening


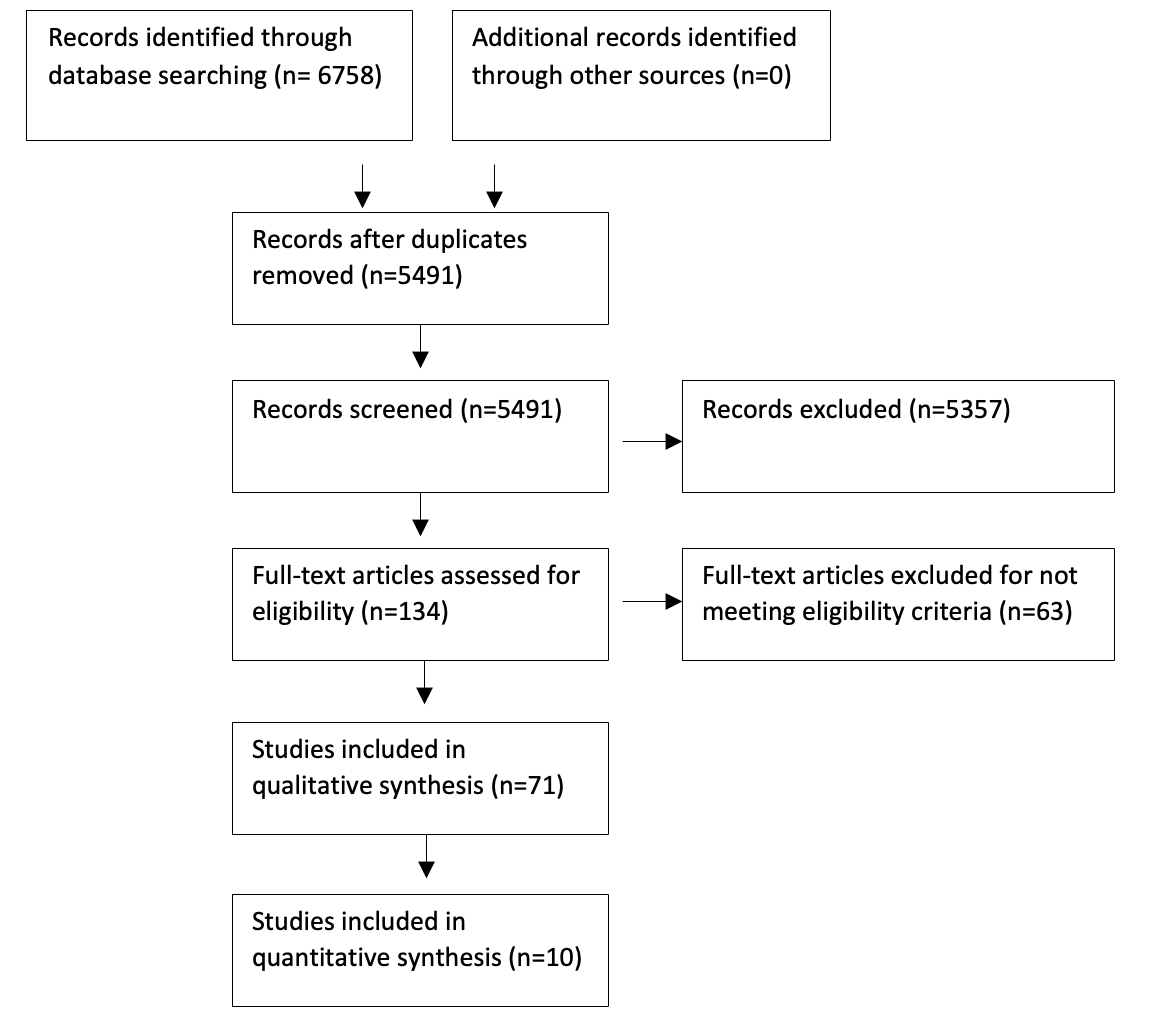


Table 1. Summary of studies

| **Author** | **Year** | **Cohort** | **Country** | **Study Design** | **Sample Size** | **Weeks Follow up** | **Mean age** | **% Female** | **Measure** |
| --- | --- | --- | --- | --- | --- | --- | --- | --- | --- |
| Zhao [41] | 2015 |  | UK | Observational | 126 | 52 | Not reported | Not reported | DAS28, HAQ, |
| Matcham [42] | 2015 |  | UK | Observational | 385 | 52 | 59 | 81 | DAS28, HAQ, ESR, TJC, SJC, PGA |
| van den Hoek [43] | 2016 |  | Netherlands | Observational | 882 | 156 | 59 | 72 | HAQ, EGA, Mortality |
| Miwa [44] | 2015 |  | Japan | Intervention | 333 | 26 | Not reported | Not reported | SDAI |
| Brown [45] | 1990 |  | USA | Observational | 387 | 182 | 53 | 75 | VAS pain |
| Kronisch [46] | 2015 | SERA | UK | Observational | 578 | 52 | 61 | 65 | HAQ |
| Corominas [47] | 2014 |  | Spain | Observational | 120 | 104 | 52 | 87 | DAS28 |
| Gwinnutt [48] | 2019 | RAMS | UK | Intervention | 463 | 52 | Not reported | 68 | Work disability (work leave, sick leave, presenteeism) |
| Rathbun [49] | 2016 | CORRONA | USA | Intervention | 1820 | 52 | 58 | 75 | CDAI |
| Matcham [33] | 2016 |  | UK | Observational | 56 | 52 | 54 | 79 | DAS28, ESR, TJC, SJC, PGA |
| Kronisch [50] | 2016 | SERA | UK | Observational | 1140 | 52 | 61 | 65 | HAQ |
| Miwa [51] | 2017 |  | Japan | Retrospective intervention | 232 | 26 | 55 | 71 | HAQ |
| Ang [52] | 2005 |  | USA | Observational | 1290 | 939 | 57 | 73 | Mortality |
| Parenti [53] | 2016 |  | USA | Retrospective intervention | 4064 | 26 | Not reported | Not reported | DAS 28, CDAI |
| Cui [54] | 2015 | OBRI | Canada | Observational | 2305 | 26 | Not reported | Not reported | DAS28, SDAI |
| Corominas [55] | 2019 |  | Spain | Intervention | 2305 | 26 | Not reported | Not reported | FACIT-F |
| Michelsen [56] | 2017 | NOR-DMARD | Norway | Intervention | 1326 | 26 | 54 | 75 | DAS28, CDAI, SDAI |
| Kleinert [57] | 2016 |  | Germany | Observational | 764 | 261 | 54 | 80 | Mortality |
| Hider [58] | 2009 |  | UK | Intervention | 160 | 52 | 56 | 72 | DAS28 |
| Bode [59] | 2012 |  | USA | Observational | 530 | 255 | 60 | 84 | Mortality |
| Leblanc-Trudeau [60] | 2015 | EUPA | Canada | Intervention | 275 | 182 | 61 | 63 | SDAI |
| McFarlane [61] | 1988 |  | Australia | Observational | 30 | 156 | 53 | 66 | DAS28 |
| Norton [62] | 2011 | ERAS | UK | Observational | 784 | 156 | 57 | 67 | DAS28, HAQ |
| Michelsen [63] | 2017 | NOR-DMARD | Norway | Intervention | 1326 | 26 | 54 | 75 | DAS28, CDAI, SDAI |
| Kuijper [27] | 2018 |  | Netherlands | Intervention | 281 | 65 | 53 | 68 | DAS28, ESR, SJC |
| Feldthusen [64] | 2016 |  | Sweden | Observational | 65 | 52 | 54 | 74 | VAS fatigue |
| Gonzalez-Lopez [38] | 2013 |  | Mexico | Observational | 123 | 52 | 44 | 73 | Sick leave |
| Tanaka [65] | 2019 |  | Japan | Intervention | 377 | 104 | Not reported | Not reported | WPAI |
| Doeglas [66] | 2004 |  | Netherlands | Observational | 264 | 156 | 53 | 65 | GARS |
| Verstappen [67] | 2007 |  | Netherlands | Intervention | 112 | 52 | 49 | 68 | HAQ |
| Hommel [68] | 1998 |  | USA | Observational | 42 | 52 | 53 | 81 | MHAQ |
| Fifield [69] | 2001 |  | USA | Observational | 415 | 417 | 58 | 83 | VAS fatigue |
| Looper [70] | 2011 | McEAR | Canada | Retrospective observational with history of depression | 104 | Medical History | 54 | 61 | HAQ |
| Nugaliyadde [71] | 2017 |  | UK | Intervention | 13 | 51 | 61 | 73 | DAS28 |
| Chung [72] | 2013 |  | Australia | Observational | 114 | 261 | Not reported | Not reported | DAS28, HAQ |
| England [73] | 2015 | BRAGGS | UK | Intervention | 1847 | 26 | 58 | 76 | DAS28 |
| Sergeant [74] | 2015 | RAMS | UK | Intervention | 460 | 26 | 60 | 73 | DAS28 |
| Morris [75] | 2011 | UCSF RA panel | USA | Observational | 1115 | 939 | 55 | 80 | HAQ |
| Van Den Hoek [76] | 2013 |  | Netherlands | Observational | 882 | 574 | 59 | 72 | HAQ, SF-36 |
| Bechman [11] | 2018 | OPTTIRA | UK | Intervention | 97 | 52 | 57 | 74 | DAS28 |
| McFarlane [77] | 1987 |  | Australia | Observational | 40 | 156 | 53 | 80 | DAS-28 |
| Odegard [36] | 2007 | EURIDISS | Norway | Observational | 238 | 521 | 52 | 74 | VAS pain |
| Vriezekolk [78] | 2010 |  | Netherlands | Intervention | 73 | 6 | 53 | 72 | AIMS, VAS pain |
| Sergeant [79] | 2016 | RAMS | UK | Intervention | 1050 | 26 | 59 | 70 | DAS28 |
| Sergeant [80] | 2018 | RAMS | UK | Intervention | 1656 | 26 | 59 | 67 | DAS28 |
| Casalla [81] | 2013 | CONAART | Argentina | Observational | 237 | 52 | 49 | 84 | DAS28 |
| Cook [82] | 2016 | NOAR | UK | Observational | 868 | 261 | 56 | 66 | SJC |
| Treharne [83] | 2008 |  | UK | Observational | 189 | 52 | 56 | 74 | VAS fatigue |
| Dobkin [84] | 2013 |  | Canada | Observational | 248 | 52 | 59 | 63 | MPQ-SF |
| Leggett [85] | 2017 | RAMS | UK | Intervention | 308 | 52 | 52 | 66 | WPS-RA |
| Dyball [32] | 2018 | BRAGGSS | UK | Intervention | 2919 | 26 | 57 | 76 | DAS28, CRP, TJC, SJC |
| Schieir [86] | 2016 | ERA | Canada | Intervention | 1595 | 52 | 54 | 72 | DAS28 |
| Parker [87] | 1992 |  | USA | Observational | 80 | 26 | 61 | 0 | SJC |
| Norton [62] | 2011 | ERAS | UK | Observational | 784 | 261 | 57 | 67 | HADS, ESR, VAS pain, TJC, SJC, Stiffness |
| Matcham [42] | 2015 | CARDERA | UK | Intervention | 467 | 104 | 54 | 68 | HAQ, ESR, SJC |
| Iannaccone [88] | 2016 |  | USA | Observational | 264 | 104 | 57 | 83 | DAS28, MHAQ, CRP |
| Schieir [89] | 2009 | McEAR | Canada | Observational | 320 | 26 | 57 | 69 | MPQ, SJC |
| Rathbun [90] | 2013 | CORRONA | USA | Observational | 4250 | 104 | Not reported | Not reported | CDAI, HAQ, CRP, ESR, VAS pain, TJC, SJC, EGA, PGA |
| Rathbun [91] | 2015 | CORRONA | USA | Observational | 12,445 | 104 | 58 | 73 | DAS28, CDAI, HAQ, CRP, ESR, TJC, SJC, EGA, PGA |
| El Miedany [92] | 2013 |  | Egypt or UK | Observational | 264 | 156 |  | 68 | DAS28, TJC, |
| Uhlig [93] | 2000 | EURIDISS | Norway | Observational | 238 | 261 | 51 | 74 | AIMS |
| Li [94] | 2019 | Truven Health Marketscan databse | USA | Observational | 46,700 | 52 | 52 | 78 | Short-term disability |
| Matcham [34] | 2014 | CARDERA | UK | Intervention | 467 | 104 | 54 | 68 | DAS28, HAQ, ESR, VAS pain, TJC, SJC, EGA |
| Van Den Hoek [95] | 2013 |  | Netherlands | Observational | 882 | 574 | 59 | Not reported | HAQ, SF-36 |
| Matcham [96] | 2014 | CARDERA | UK | Intervention | 379 | 104 | 54 | 68 | SJC |
| Crotty [97] | 1994 |  | Australia | Observational | 75 | 191 | 42 | 100 | HAQ |
| Overman [30] | 2011 |  | Netherlands | Intervention | 545 | 26 | 56 | 69 | TJC |
| Matcham [28] | 2018 | BSRBR-RA | UK | Intervention | 18,421 | 52 | 56 | 76 | DAS28, ESR, SJC, EGA |
| Euesden [98] | 2017 | CARDERA | UK | Intervention | 520 | 104 | 55 | 69 | DAS28, HAQ, ESR, VAS pain, TJC, SJC, EGA |
| Smedstad [99] | 1997 | EURIDISS | Norway | Observational | 238 | 104 | 52 | 73 | HAQ, VAS pain |
| Karpouzas [100] | 2017 | UCLA RA cohort | USA | Observational | 156 | 52 | 52 | 89 | HAQ |

Table 2. Vote counting

| **Outcome** | **Number of studies** | **Vote count for direction of effect of affective distress on outcomes** | | |
| --- | --- | --- | --- | --- |
|  |  | Negative effect | Positive effect | No effect/  unclear |
| **Disease Activity**  Disease Activity Score (DAS), including remission  Simple Disease Activity Index (SDAI)  Clinical Disease Activity Index (CDAI) | 34  26  4  4 | 1  0  0 | 12  3  3 | 13  1  1 |
| **Disability**  Health Assessment Questionnaire (HAQ)  Arthritis Impact Measurement Scale (AIMS)  Activities of Daily Living (ADL)  Groningen Activity Restriction Scale (GARS)  Modified Health Assessment Questionnaire (MHAQ)  Short-term disability days | 23  16  2  1  1  2  1  1 | 0  0  0  0  0  0 | 7  1  0  0  1  1 | 9  1  1  1  1  0 |
| **Pain**  Visual Analog Scale (VAS)  Arthritis Impact Measurement Scales (AIMS)  McGill Pain Questionnaire (MPQ) | 12  9  1  2 | 0  0  0 | 3  1  1 | 6  0  1 |
| **C-reactive protein (CRP)** | 3 | 0 | 0 | 3 |
| **Erythrocyte Sedimentation Rate (ESR)** | 8 | 0 | 0 | 8 |
| **Tender Joint Count (TJC)** | 7 | 0 | 5 | 2 |
| **Swollen** Joint Count (**SJC)** | 7 | 0 | 5 | 2 |
| **Physician Global Assessment (EGA)** | 4 | 0 | 3 | 1 |
| **Patient Global Assessment (PGA)** | 3 | 0 | 2 | 0 |
| **Work disability**  Presenteeism (WPS-RA)  Sick leave  Work productivity (WPAI)  Left Work | 4  2  2  1  1 | 1  0  1  0 | 0  2  0  0 | 1  0  0  1 |
| **Mortality** | 4 | 0 | 4 | 0 |
| **Stiffness** | 1 | 0 | 0 | 1 |
| **Fatigue**  Visual Analog Scale (VAS)  Functional Assessment of Chronic Illness Therapy (FACIT-F) | 4  3  1 | 0  0 | 1  1 | 2  0 |

Table 3. Quality Assessment using the Newcastle-Ottowa Scale

| **Study** | **Year** | **Selection** | | | | **Comparability** | **Outcome** | | | **Quality Total** |
| --- | --- | --- | --- | --- | --- | --- | --- | --- | --- | --- |
|  |  | Representativeness | Non-exposed  cohort | Ascertainment of exposure | Outcome did not exist at start | Comparability of Design or Analysis  (2 stars) | Assessment of outcome | Follow up long enough | Adequacy of follow ups |  |
| Zhao | 2015 | 1 | 1 | 0 | 1 | 1 | 0 | 1 | 1 | 6 |
| Matcham | 2015 | 1 | 1 | 1 | 1 | 1 | 1 | 1 | 0 | 7 |
| van den Hoek | 2016 | 1 | 1 | 0 | 1 | 2 | 0 | 1 | 0 | 6 |
| Miwa | 2015 | 1 | 1 | 1 | 1 | 0 | 1 | 1 | 0 | 6 |
| Brown | 1990 | 1 | 1 | 0 | 1 | 0 | 0 | 1 | 1 | 5 |
| Kronisch | 2015 | 1 | 1 | 1 | 1 | 1 | 1 | 1 | 1 | 8 |
| Corominas | 2014 | 1 | 1 | 1 | 1 | 0 | 1 | 1 | 0 | 6 |
| Gwinnutt | 2019 | 1 | 1 | 1 | 1 | 1 | 1 | 1 | 0 | 7 |
| Rathbun | 2016 | 1 | 1 | 0 | 1 | 2 | 1 | 1 | 1 | 8 |
| Matcham | 2016 | 1 | 1 | 0 | 1 | 1 | 0 | 1 | 1 | 6 |
| Kronisch | 2016 | 1 | 1 | 1 | 1 | 1 | 1 | 1 | 1 | 8 |
| Miwa | 2017 | 1 | 1 | 1 | 1 | 0 | 1 | 1 | 0 | 6 |
| Ang | 2005 | 1 | 1 | 0 | 1 | 2 | 0 | 1 | 0 | 6 |
| Parenti | 2016 | 1 | 1 | 1 | 1 | 0 | 1 | 1 | 0 | 6 |
| Cui | 2015 | 1 | 1 | 0 | 1 | 1 | 1 | 1 | 0 | 6 |
| Corominas | 2019 | 1 | 1 | 1 | 1 | 2 | 1 | 1 | 1 | 9 |
| Michelsen | 2017 | 1 | 1 | 0 | 1 | 1 | 0 | 1 | 0 | 5 |
| Kleinert | 2016 | 1 | 1 | 0 | 1 | 0 | 1 | 1 | 0 | 5 |
| Hider | 2009 | 1 | 1 | 1 | 1 | 0 | 1 | 1 | 1 | 7 |
| Bode | 2012 | 1 | 1 | 1 | 1 | 1 | 1 | 1 | 0 | 7 |
| Leblanc-Trudeau | 2015 | 1 | 1 | 1 | 1 | 1 | 1 | 1 | 1 | 8 |
| McFarlane | 1988 | 1 | 1 | 0 | 1 | 1 | 0 | 1 | 1 | 6 |
| Norton | 2011 | 1 | 1 | 0 | 1 | 1 | 0 | 1 | 0 | 5 |
| Michelsen | 2017 | 1 | 1 | 0 | 1 | 1 | 0 | 1 | 0 | 5 |
| Kuijper | 2018 | 1 | 1 | 0 | 1 | 1 | 0 | 1 | 0 | 5 |
| Feldthusen | 2016 | 1 | 1 | 0 | 1 | 1 | 0 | 1 | 0 | 5 |
| Gonzalez-Lopez | 2013 | 1 | 1 | 1 | 1 | 1 | 1 | 1 | 1 | 8 |
| Tanaka | 2019 | 1 | 1 | 0 | 1 | 0 | 0 | 1 | 1 | 5 |
| Doeglas | 2004 | 1 | 1 | 1 | 1 | 1 | 1 | 1 | 1 | 8 |
| Verstappen | 2007 | 1 | 1 | 0 | 1 | 1 | 0 | 1 | 0 | 5 |
| Hommel | 1998 | 1 | 1 | 0 | 1 | 0 | 0 | 1 | 0 | 4 |
| Fifield | 2001 | 1 | 1 | 0 | 1 | 0 | 0 | 1 | 1 | 5 |
| Looper | 2011 | 1 | 1 | 1 | 1 | 1 | 1 | 1 | 0 | 7 |
| Nugaliyadde | 2017 | 1 | 1 | 0 | 1 | 0 | 0 | 1 | 0 | 4 |
| Chung | 2013 | 1 | 1 | 0 | 1 | 1 | 0 | 1 | 0 | 5 |
| England | 2015 | 1 | 1 | 0 | 1 | 0 | 0 | 1 | 0 | 4 |
| Sergeant | 2015 | 1 | 1 | 0 | 1 | 0 | 0 | 1 | 0 | 4 |
| Morris | 2011 | 1 | 1 | 0 | 1 | 1 | 0 | 1 | 0 | 5 |
| Van Den Hoek | 2013 | 1 | 1 | 0 | 1 | 0 | 0 | 1 | 0 | 4 |
| Bechman | 2018 | 1 | 1 | 0 | 1 | 1 | 0 | 1 | 0 | 5 |
| McFarlane | 1987 | 1 | 1 | 0 | 1 | 0 | 0 | 1 | 0 | 4 |
| Odegard | 2007 | 1 | 1 | 0 | 1 | 1 | 0 | 1 | 1 | 6 |
| Vriezekolk | 2010 | 1 | 1 | 0 | 1 | 0 | 0 | 1 | 1 | 5 |
| Sergeant | 2016 | 1 | 1 | 0 | 1 | 1 | 0 | 1 | 0 | 5 |
| Sergeant | 2018 | 1 | 1 | 1 | 1 | 1 | 1 | 1 | 1 | 8 |
| Casalla | 2013 | 1 | 1 | 0 | 1 | 1 | 0 | 1 | 1 | 6 |
| Cook | 2016 | 1 | 1 | 1 | 1 | 1 | 1 | 1 | 0 | 7 |
| Treharne | 2008 | 1 | 1 | 1 | 1 | 1 | 1 | 1 | 0 | 7 |
| Dobkin | 2013 | 1 | 1 | 0 | 1 | 1 | 0 | 1 | 1 | 6 |
| Leggett | 2017 | 1 | 1 | 0 | 1 | 1 | 0 | 1 | 0 | 5 |
| Dyball | 2018 | 1 | 1 | 0 | 1 | 1 | 0 | 1 | 0 | 5 |
| Schieir | 2016 | 1 | 1 | 0 | 1 | 1 | 0 | 1 | 0 | 5 |
| Parker | 1992 | 1 | 1 | 1 | 1 | 0 | 1 | 1 | 0 | 6 |
| Norton | 2011 | 1 | 1 | 0 | 1 | 1 | 0 | 1 | 0 | 5 |
| Matcham | 2015 | 1 | 1 | 0 | 1 | 1 | 0 | 1 | 1 | 6 |
| Iannaccone | 2016 | 1 | 1 | 0 | 1 | 1 | 0 | 1 | 0 | 5 |
| Schieir | 2009 | 1 | 1 | 0 | 1 | 0 | 0 | 1 | 0 | 4 |
| Rathbun | 2013 | 1 | 1 | 0 | 1 | 0 | 0 | 1 | 0 | 4 |
| Rathbun | 2015 | 1 | 1 | 1 | 1 | 2 | 1 | 1 | 0 | 8 |
| El Miedany | 2013 | 1 | 1 | 0 | 1 | 0 | 0 | 1 | 0 | 4 |
| Uhlig | 2000 | 1 | 1 | 0 | 1 | 0 | 0 | 1 | 1 | 5 |
| Li | 2019 | 1 | 1 | 0 | 1 | 0 | 0 | 1 | 0 | 4 |
| Matcham | 2014 | 1 | 1 | 0 | 1 | 1 | 0 | 1 | 0 | 5 |
| Van Den Hoek | 2013 | 1 | 1 | 0 | 1 | 2 | 0 | 1 | 0 | 6 |
| Matcham | 2014 | 1 | 1 | 0 | 1 | 1 | 0 | 1 | 0 | 5 |
| Crotty | 1994 | 1 | 1 | 0 | 1 | 1 | 0 | 1 | 0 | 5 |
| Overman | 2011 | 1 | 1 | 0 | 1 | 2 | 0 | 1 | 0 | 6 |
| Matcham | 2018 | 1 | 1 | 1 | 1 | 1 | 1 | 1 | 0 | 7 |
| Euesden | 2017 | 1 | 1 | 0 | 1 | 1 | 0 | 1 | 0 | 5 |
| Smedstad | 1997 | 1 | 1 | 0 | 1 | 0 | 0 | 1 | 0 | 4 |
| Karpouzas | 2017 | 1 | 1 | 0 | 1 | 0 | 0 | 1 | 0 | 4 |
